# Joint QTL mapping and transcriptome sequencing analysis reveal candidate flowering time genes in *Brassica napus* L

**DOI:** 10.1186/s12864-018-5356-8

**Published:** 2019-01-09

**Authors:** Hongju Jian, Aoxiang Zhang, Jinqi Ma, Tengyue Wang, Bo Yang, Lan Shuan Shuang, Min Liu, Jiana Li, Xinfu Xu, Andrew H. Paterson, Liezhao Liu

**Affiliations:** 1 Chongqing Engineering Research Center for Rapeseed, College of Agronomy and Biotechnology, Southwest University, Academy of Agricultural Sciences, Chongqing, 400715 China; 2 Plant Genome Mapping Laboratory, University of Georgia, Athens, GA 30605 USA

**Keywords:** Flowering time, QTL, Gene expression, RNA-Seq, *Brassica napus*

## Abstract

**Background:**

Optimum flowering time is a key agronomic trait in *Brassica napus*. To investigate the genetic architecture and genetic regulation of flowering time in this important crop, we conducted quantitative trait loci (QTL) analysis of flowering time in a recombinant inbred line (RIL) population, including lines with extreme differences in flowering time, in six environments, along with RNA-Seq analysis.

**Results:**

We detected 27 QTLs distributed on eight chromosomes among six environments, including one major QTL on chromosome C02 that explained 11–25% of the phenotypic variation and was stably detected in all six environments. RNA-Seq analysis revealed 105 flowering time-related differentially expressed genes (DEGs) that play roles in the circadian clock/photoperiod, autonomous pathway, and hormone and vernalization pathways. We focused on DEGs related to the regulation of flowering time, especially DEGs in QTL regions.

**Conclusions:**

We identified 45 flowering time-related genes in these QTL regions, eight of which are DEGs, including key flowering time genes *PSEUDO RESPONSE REGULATOR 7* (*PRR7*) and *FY* (located in a major QTL region on C02). These findings provide insights into the genetic architecture of flowering time in *B. napus*.

**Electronic supplementary material:**

The online version of this article (10.1186/s12864-018-5356-8) contains supplementary material, which is available to authorized users.

## Background

In flowering plants, the transition from the vegetative stage to the reproductive stage helps to ensure reproductive success, including successful seed production [1]. This trait is especially important in crop plants, as it can determine crop cultivation ranges and ensure high productivity. Thus, flowering time is a vital trait that is a target of selection during crop breeding. Flowering time is sensitive to various environmental signals (such as day length and temperature) and endogenous signals (e.g., developmental stage and age) [2, 3]. To date, much is known about candidate genes controlling flowering time in *Arabidopsis thaliana*. More than 300 flowering time genes have been identified, and several key regulators that function in pathways that control flowering time have been detected [4, 5]. Six major pathways control flowering time in Arabidopsis: vernalization, the photoperiod/circadian clock, and the ambient temperature, gibberellin, autonomous, and endogenous pathways [1, 4, 6–8]. In Arabidopsis, *FLOWERING LOCUS C* (*FLC*) and *FRIGIDA* (*FRI*) are key genes in the vernalization response, whereas *CONSTANS* (*CO*) functions in the response to photoperiod [9, 10]. *FLOWERING LOCUS T* (*FT*) encodes a mobile signal long described as “florigen”, which functions as a central floral integrator in the control of flowering [11].

Oilseed rape (*Brassica napus* L., also known as rapeseed or canola) is one of the most important oil crops worldwide. Many important and complex agronomic traits such as yield [12], plant height [13], oil content [14], seed weight [15], and flowering time [16] have been mapped in this crop. Flowering time in rapeseed not only has a crucial impact on yield, but it also influences the sowing time of other rotation crops [16]. Quantitative trait locus (QTL) analysis and genome-wide associated mapping (GWAS) have been used to identify candidate flowering time genes in oilseed rape. Many QTLs related to flowering time have been identified in this crop. For example, one major QTL was identified that explains 50% of the total phenotypic variation for flowering time in *B. napus*. This QTL is related to *VFN2*, a major vernalization-responsive flowering time gene in Arabidopsis [17]. Raman et al. (2013) performed QTL analysis for flowering time using a doubled haploid (DH) population [18]. Liu et al. (2016) identified 22 QTLs (including four major QTLs) for flowering time in *B. napus* using a DH population [19]. GWAS was also recently used to screen for candidate flowering time genes in *B. napus*. Xu et al. (2016) identified 41 SNPs associated with flowering time using GWAS of 523 *B. napus* cultivars [20]. Raman et al. (2016) obtained 69 SNP markers associated with flowering time using GWAS approaches and detected several candidate flowering time genes, such as *FT*, *FRUITFUL*, *FLC*, *CO*, *FRI*, and *PHYTOCHROME B*, within 20 Kb regions [21]. QTLs or genes have also been identified in other *Brassica* crops, such as *B. rapa* [22, 23] and *B. oleracea* [24].

Although much effort has focused on investigating flowering time, stable QTLs for this trait have not yet been identified, and global transcriptome analysis of different rapeseed genotypes has not yet been performed. Therefore, in this study, we performed joint QTL mapping and RNA-Seq analysis to uncover the genetic architecture of flowering time in *B. napus*.

## Materials and methods

### Plant materials and growth conditions

A recombinant inbred line (RIL) population consisting of 172 lines was constructed from a cross between GH06 (female parent, late flowering, semi-winter) and P174 (male parent, early flowering, semi-winter). The GH06 × P174 RIL population was previously used to map seed fiber content in oilseed rape [25]. The population was obtained from Chongqing Engineering Research Center for Rapeseed,Southwest University.

The flowering time trait was evaluated in six environments (the temperature data in each environment was shown in Additional file 1: Table S1), including Giessen (E8.76/N50.56), Germany in 2009 (09Gi) and Beibei (E106.26/N29.82), Chongqing, China in 2012–2016 (12Cq, 13Cq, 14Cq, 15Cq, and 16Cq, respectively). In Giessen, the seeds were sown directly in the spring of 2009. In Chongqing, seeds from the RILs and the parental lines were sown in nursery beds on September 18, 2012, 2013, 2014, 2015, and 2016 and transplanted to the field one month later. Each line of the RIL population was grown in a 4.5 m^2^ (1.5 × 3) plot with 80–90 plants (in Giessen environment) or 50–60 plants (in Chongqing environment). Flowering time data were recorded for each line from the sowing day to the day when 50% of the plants showed the first blooming floret.

### Genetic and QTL mapping

A high-density SNP genetic map was constructed using the *Brassica* 60 K BeadChip Array [25]. A genetic map containing 2795 SNP markers with a mean distance of 0.66 cM between adjacent SNP markers was used for QTL mapping.

Windows QTL Cartographer version 2.5 with default settings was used to detect QTLs for flowering time via the composite interval mapping method [26]. The logarithm of the odds (LOD) threshold for detecting a significant QTL was established by permutation analysis with 1000 permutations. The linkage map and QTL position was generated using MapChart software [27].

To screen candidate genes in QTL regions, following procedures were conducted: (1) 1-LOD likelihood intervals surrounding the peak of the QTL likelihood plot were regarded as the QTL interval; (2) Ten SNPs located within and at each end of each interval were considered, selecting the SNP with either the largest or smallest physical distance at each end to maximize the physical size of the region, based on previously published physical locations of each SNP [28]; (3), Genes located in the intervals were selected as candidate genes based on published annotation of the *B. napus* genome [29].

### RNA isolation and transcriptome sequencing

Five early-flowering lines (marked “E”) and five late-flowering lines (marked “L”) were selected from the RIL population based on the flowering time in six environments. To detect candidate genes involved in regulating the days to flowering, shoot tissues (S) and leaves (L) were collected from both E and L lines at 10 o’clock am in the vegetative stage at 20 weeks after germination in 16Cq environment. For both the E and L lines, shoot tissues (ES and LS) or leaves (EL and LL) from five lines were pooled, immediately frozen in liquid nitrogen, and stored at − 80 °C until use.

Total RNA was isolated from each sample using a Plant RNA Mini Kit (Tiangen, Inc., China) according to the manufacturer’s protocol. Four cDNA libraries were constructed and RNA-Seq was performed on an Illumina HiSeq 2500 platform by Novogene Bioinformatics Technology Co. Ltd. (Beijing, China) according to the manufacturer’s instructions. Moreover, these paired end sequencing reads were immediately uploaded to NCBI with accession number SRP108958.

### RNA sequencing data analysis

High-quality reads were obtained after the adapter sequences and low quality sequences were filtered out from the raw data using the NGS QC toolkit [30]. The clean reads were mapped to the *B. napus* genome (http://www.genoscope.cns.fr/brassicanapus/data/) using TopHat v2.0.11. Unique reads were further analyzed and gene expression levels were calculated using Cufflinks v2.2.0 [31]. Gene expression levels were estimated by the FPKM (fragments per kilobase of exon per million mapped fragments) method, and DEGs were identified using the criteria FDR ≤ 0.01 and |log_2_ (FPKM _early_/FPKM _late_)| ≥ 1.

To further investigate the potential functions of the DEGs, KEGG enrichment analysis was performed using the KOBAS2.0 website (http://kobas.cbi.pku.edu.cn/home.do).

### Identification of *B. napus* homologs of flowering time-related genes

To discover flowering time genes in *B. napus*, 306 flowering-time related (FTR) genes in *A. thaliana* were downloaded from the Flowering Interactive Database (http://www.phytosystems.ulg.ac.be/florid/). Homologs of these genes in *B. napus* were identified by BLASTN analysis against the *B. napus* reference genome. Top hits with E-values ≤1*E*^*− 20*^ and identity ≥80% were used to screen for the corresponding homologous genes.

### qRT-PCR confirmation of RNA-Seq data

To confirm the RNA-Seq data and the DEGs identified in the early- and late-flowering lines, 47 genes were subjected to qRT-PCR analysis. One microgram of total RNA per sample (the same samples used for RNA-seq) was used to synthesize cDNA using the M-MuLV RT kit (Takara Biotechnology, Japan) according to the manufacturer’s instructions (TransGen, China). The qPCR was performed as described previously [32]. *BnACTIN7* and *BnUBC21* were used as internal controls, and the 2^-ΔΔCt^ method was used to evaluate relative gene expression levels. The gene-specific primers are shown in Additional file 2: Table S2. Each PCR was performed with three technical replicates.

## Results

### Analysis of flowering time in six environments

**Fig. 1:**
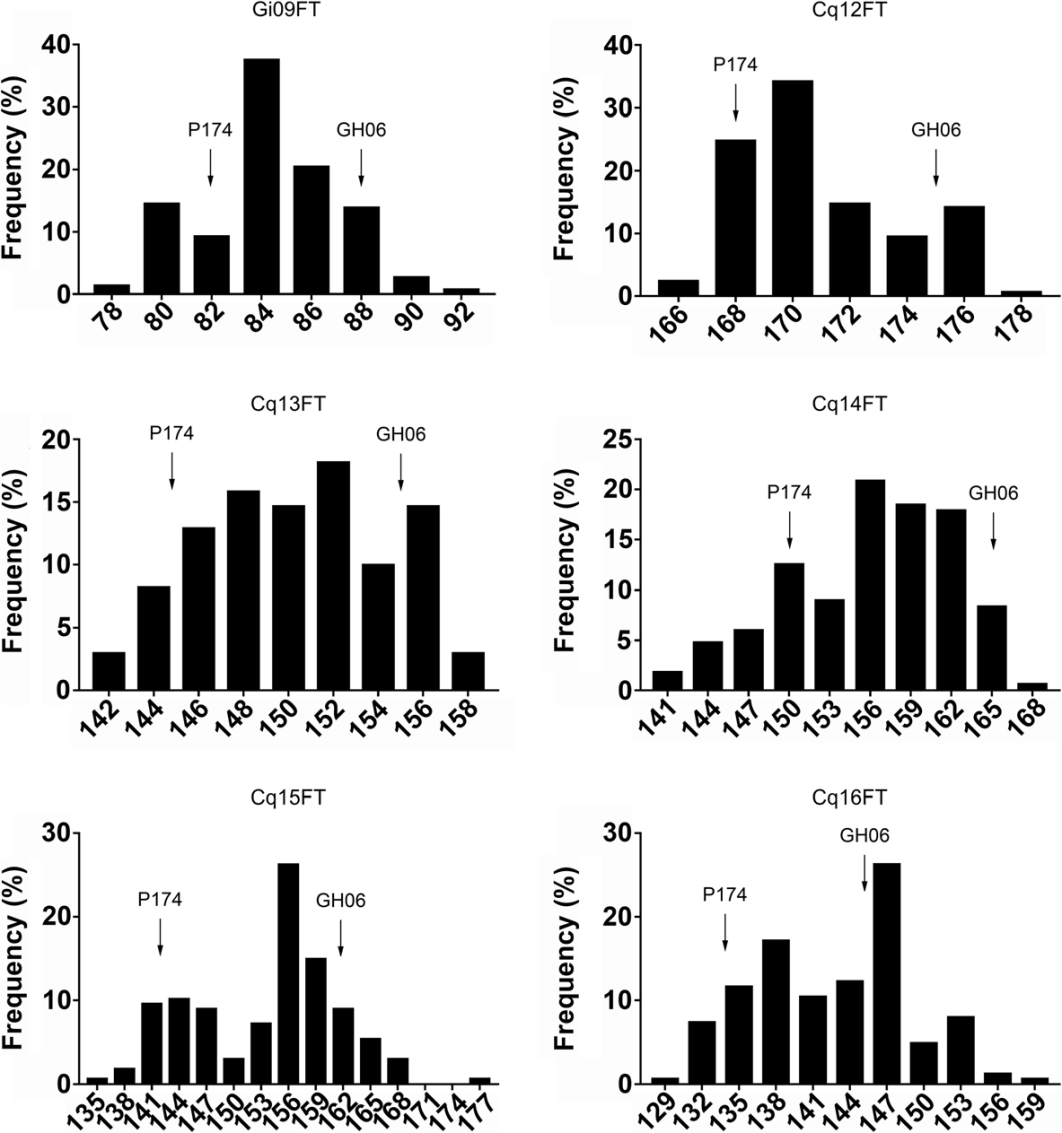
Frequency distribution of the flowering time trait in RILs grown in six different environments. 09Gi: Germany in 2009; 12Cq: Chongqing in 2012; 13Cq: Chongqing in 2013; 14Cq: Chongqing in 2014; 15Cq: Chongqing in 2015; 16Cq: Chongqing in 2016; P174: male parent; GH06: female parent

We analyzed flowering time traits in a population of 172 RILs. The flowering time values of the two parental lines, as well as the mean, maximum, and minimum values of the RIL population for flowering time in six environments, were summarized in Table 1. The transgressive segregation of flowering time traits in all six environments was shown in Fig. 1. We detected a great difference between the two parental lines and within the RIL populations. The correlation coefficients of flowering time among the six environments are shown in Additional file 3: Table S3. Our results indicate that flowering time is positively and significantly correlated among the six environments (r^2^ = 0.255–0.766, *P* < 0.01). The correlation between the German and the Chinese locations (r^2^ < 0.4) are lower than among the Chinese environments (r^2^ > 0.6) because of the great difference between German environment and Chinese environment.

**Table 1:** Phenotypic variation in flowering time in the RILs and their parents

Environment	Parents		RIL population			
	GH06	P174	Minimum	Maximum	Mean	Std. Deviation
Gi09FT	88	82	78	92	83.76	2.555
Cq12FT	175	168	165	177	170.13	2.775
Cq13FT	155	145	142	158	149.81	4.173
Cq14FT	165	150	142	167	156.01	6.014
Cq15FT	161	142	134	178	153.80	7.968
Cq16FT	146	134	130	158	142.74	6.299

*09Gi* Germany in 2009, *12Cq* Chongqing in 2012, *13Cq* Chongqing in 2013, *14Cq* Chongqing in 2014, *15Cq* Chongqing in 2015, *16Cq* Chongqing in 2016

### Mapping of QTLs for flowering time in six environments

**Fig. 2:**
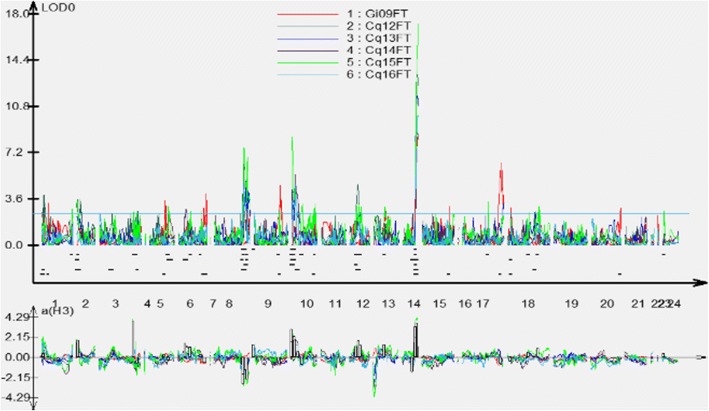
Graphs of QTLs for flowering time in an RIL population throughout the genome in plants grown in six different environments. 09Gi: Germany in 2009; 12Cq: Chongqing in 2012; 13Cq: Chongqing in 2013; 14Cq: Chongqing in 2014; 15Cq: Chongqing in 2015; 16Cq: Chongqing in 2016

**Fig. 3:**
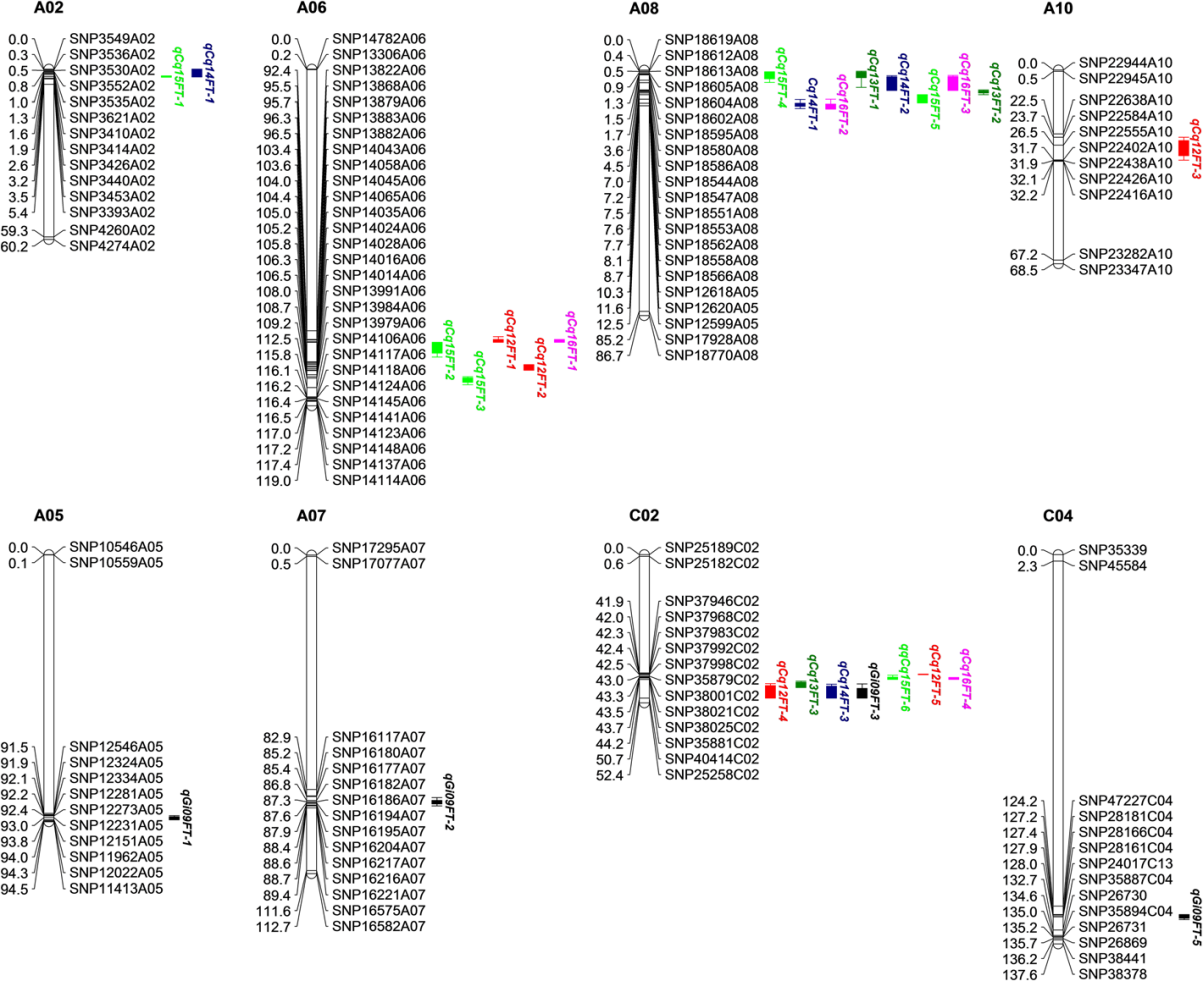
Locations of significant QTLs for flowering time on a high-density SNP map. For simplicity, only markers in QTL confidence intervals, along with the two terminal markers at each end of each QTL-containing chromosome, are shown. Full map data are provided in Liu et al. (2013). 09Gi: Germany in 2009; 12Cq: Chongqing in 2012; 13Cq: Chongqing in 2013; 14Cq: Chongqing in 2014; 15Cq: Chongqing in 2015; 16Cq: Chongqing in 2016

We detected 27 QTLs distributed on eight chromosomes in the six environments, with 5.2–25.1% phenotypic variation (PV) and additive effects ranging from − 2.83 to 3.64 (Table 3). Among these QTLs, 1–8 QTLs were detected on eight chromosomes and 3–6 QTLs were identified in each environment (Figs. 2 and 3, Table 2). The values of the additive effects of QTLs on A05, A06, A07, and C04 were negative, whereas those of QTLs on A02, A08, A10, and C02 were positive, indicating that the genetic background of the female parent causes later flowering and that of the male parent causes earlier flowering. By aligning SNP markers in these regions, we identified the physical locations of these QTL regions in the *B. napus* genome, leading to the detection of 3436 genes (Table 3, Additional file 4: Table S4).

**Table 2:** Significant QTLs associated with flowering time in the RIL population

QTL names	Trait	Chromosome	Position (cM)	Additive	LOD	QTL region (cM)	R^2^
**qFTA02**	Cq14FT	A02	2.01	1.55	3.28	0–2.8	0.05
	Cq15FT	A02	2.61	1.84	3.53	2.4–2.9	0.06
qFTA05	Gi09FT	A05	93.01	−0.59	4.02	92.7–93.8	0.07
**qFTA06–1**	Cq12FT	A06	96.31	−0.95	6.10	94.5–96.5	0.12
	Cq15FT	A06	97.51	−2.83	7.63	96.5–101.7	0.10
	Cq16FT	A06	96.31	−1.85	4.11	95.5–96.5	0.07
qFTA06–2	Cq12FT	A06	105.81	−0.76	4.16	104.4–106.4	0.06
qFTA06–3	Cq15FT	A06	109.21	−2.34	6.87	108.7–111.5	0.07
qFTA07	Gi09FT	A07	87.31	−0.78	4.67	85.8–88.7	0.09
**qFTA08–1**	Cq13FT	A08	1.31	1.16	4.33	0.4–6.1	0.07
	Cq15FT	A08	1.31	2.99	8.48	0.6–4.4	0.14
	Cq14FT	A08	2.71	1.86	4.30	2–7.2	0.09
	Cq16FT	A08	2.71	1.68	3.50	1.9–7.2	0.06
	Cq13FT	A08	7.61	1.00	3.09	7–8.7	0.05
**qFTA08–2**	Cq15FT	A08	9.71	2.25	3.98	8.7–11.3	0.07
	Cq14FT	A08	12.51	1.90	5.52	10.4–13.5	0.11
	Cq16FT	A08	12.71	1.83	4.72	10.3–13.5	0.09
	Cq12FT	A10	27.51	0.83	4.73	23.7–31.8	0.07
**qFTC02–1**	Cq12FT	C02	42.41	1.12	7.12	42.3–42.5	0.17
	Cq15FT	C02	43.71	3.26	8.46	42.7–44.2	0.16
	Gi09FT	C02	43.71	0.87	4.82	42.5–44.2	0.11
	Cq16FT	C02	43.71	2.16	5.65	43.5–44.2	0.12
**qFTC02–2**	Cq13FT	C02	46.21	2.06	11.70	44.8–47.1	0.21
	Cq14FT	C02	49.11	3.64	8.91	45.9–50.7	0.19
	Cq12FT	C02	48.11	1.43	13.30	45.7–50.7	0.25
	Gi09FT	C02	50.11	1.14	8.57	45.8–50.7	0.19
qFTC04	Gi09FT	C04	127.91	−0.68	6.23	127.2–129	0.09

*09Gi* Germany in 2009, *12Cq* Chongqing in 2012, *13Cq* Chongqing in 2013, *14Cq* Chongqing in 2014, *15Cq* Chongqing in 2015, *16Cq* Chongqing in 2016. Stable QTL were represented bold

**Table 3:** Number of FTR genes in QTL regions

Name	QTL regions	Gd. (cM)	Pl. (bp)	No. of genes	No. of FTR genes
QTL-A02	SNP3549A02-SNP3440A02	0–3.2	A02: 19650440–20,741,204	148	0
QTL-A06	SNP13822A06-SNP13882A06	92.398–96.47	A06: 7212328–21,686,640	1909	33
QTL-A07	SNP16177A07-SNP16216A07	85.8–88.7	A07: 19645576–19,981,239	55	0
QTL-A08	SNP18612A08-SNP18586A08	0.408–4.458	A08: 18395488–18,767,730	89	2
QTL-A10	SNP22584A10-SNP22402A10	23.7–31.736	A10: 13337887–14,487,871	255	3
QTL-C02	SNP35881C02-SNP25258C02	44.191–50.702	CO2: 15599–5,545,697	980	7

In total, 3436 genes were detected in QTL regions, including 45 FTR genes. No FTR genes were detected in QTL regions on chromosome A02 or A07

### Illumina sequencing and global analysis of gene expression

To gain insights into the transcriptomic changes in the early- and late-flowering lines, we performed RNA-Seq analysis of four samples, representing leaf and shoot tissues from early- and late-flowering lines. After removing 0.82–1.41% of the sequences, including low-quality reads and adapter sequences, 29.87 Gb of clean data were obtained and used for quantitative analysis of gene expression. We mapped these clean reads to the reference *B. napus* genome using TopHat software; 69.75–71.62% of the clean reads were mapped to the genome, including 62.16–65.46% and 5.54–8.43% uniquely mapped and multi-mapped reads, respectively, while the remaining reads (28.38–30.25%) were unmapped (Table 4).

**Table 4:** Summary of read numbers from the RNA-Seq data for the four samples

Sample ID	Total Reads	Mapped Reads	Unique Mapped Reads	Multiple Mapped Reads	Unmapped Reads
EL	50,590,768	35,840,072 (70.84%)	31,573,071 (62.41%)	4,267,001 (8.43%)	14,750,696 (29.16%)
LL	46,020,194	32,432,370 (70.47%)	28,605,892 (62.16%)	3,826,478 (8.31%)	13,588,824 (29.53%)
ES	51,678,990	36,044,431 (69.75%)	33,179,361 (64.20%)	2,865,070 (5.54%)	15,634,559 (30.25%)
LS	51,644,744	36,989,817 (71.62%)	33,808,757 (65.46%)	3,181,060 (6.16%)	14,654,927 (28.38%)

*EL* leaves of early-flowering bulks, *LL* leaves of late-flowering bulks, *ES* shoots of late-flowering bulks, *LS* shoots of late-flowering bulks

**Fig. 4:**
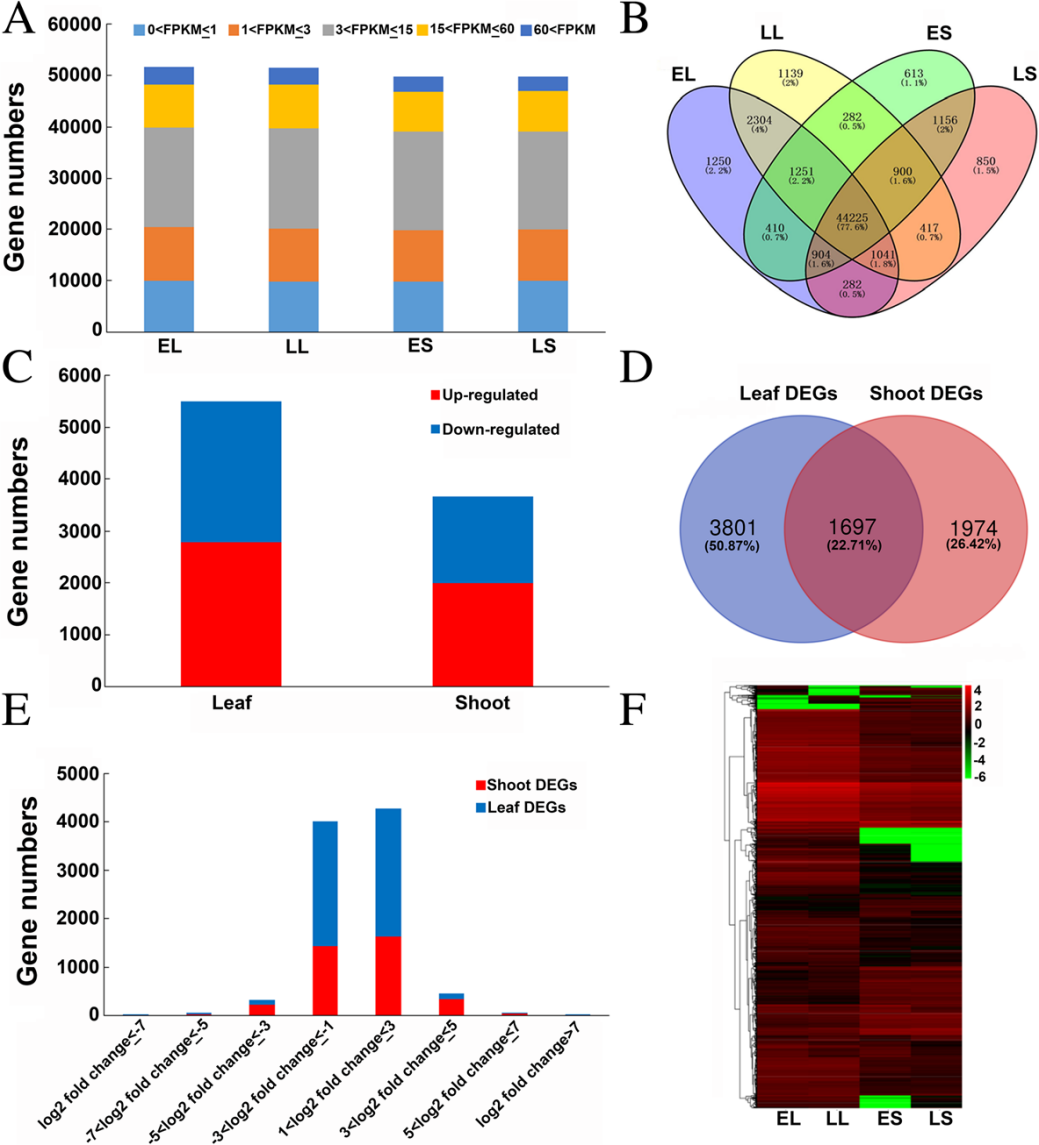
Gene expression profiles and DEGs identified between early- and late-flowering bulks. **a** Statistical analysis of data from the four samples, i.e. early- and late-flowering bulks in leaf and shoot tissues: **b** Venn diagram of the number of genes detected in the four samples. **c** Identification of DEGs in leaf and shoot tissues between early- and late-flowering bulks. **d** Venn diagram of DEGs in leaf and shoot tissues. **e** Fold changes in the expression of DEGs detected between early- and late-flowering bulks in leaf and shoot tissues, respectively. **f** Heat map of gene expression levels in the four samples. Fold change: FPKM _early_/FPKM _late_

Using FPKM analysis, 58,266 genes with values of FPKM≥0.1 were identified in the four libraries. Additionally, 19.08–19.97% of the genes in the four libraries had very low expression levels (FPKM< 1.0), 20.08–20.23% had low expression levels (1.0 ≤ FPKM< 3.0), 37.60–38.79% had moderate expression levels (3.0 ≤ FPKM< 15.0), 15.54–16.15% had high expression levels (15.0 ≤ FPKM< 60.0), and 5.81–6.76% had very high expression levels (FPKM≥60.0) (Fig. 4a). The distribution of expressed genes in the four libraries is shown in Fig. 4b 44,225 (76.0%) genes were expressed in all four libraries, and 613–1250 genes were uniquely expressed in one of the four libraries (Fig. 4b).

### Transcriptome differences between early- and late-flowering lines

To identify important genes responsible for flowering time variation, we selected 5498 and 3671 significant DEGs based on the criteria |log_2_ (FPKM _early_/FPKM _late_)| ≥ 0.58 and FDR ≤ 0.01 in leaf and shoot tissues, respectively. Of the 5498 DEGs in leaves, 2707 (49.2%) genes were downregulated and 2791 (50.8%) were upregulated. A total of 3671 DEGs, including 1673 (45.6%) downregulated genes and 1998 (54.4%) upregulated genes, were detected in shoot tissues (Fig. 4c). In addition, 1697 DEGs were common to both leaf and shoot tissues, whereas 3801 and 1974 DEGs were specific to leaf and shoot tissue, respectively (Fig. 4d). Moreover, the fold changes in the expression (up- or downregulation) of most DEGs in both leaf and shoot tissues were approximately 2–8 (Fig. 4e). We constructed a heatmap of the expression patterns of these DEGs in the four samples using MeV4.9 software (Fig. 4f).

**Fig. 5:**
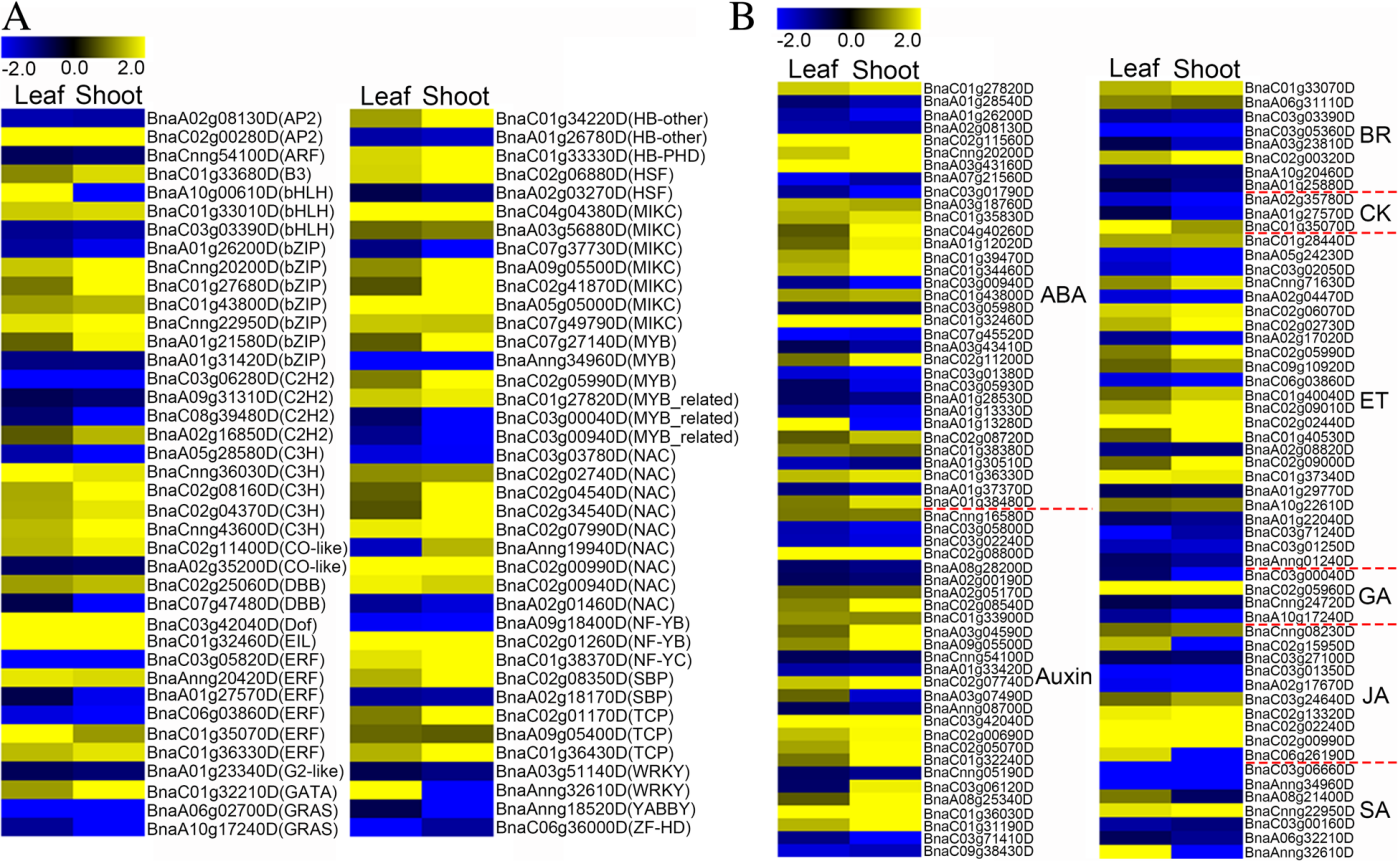
Heat maps of TF (**a**) and hormone-related (**b**) genes identified among the DEGs. ABA: abscisic acid; BR: brassinolide; CK: cytokinin; ET: ethylene; GA: gibberellin; JA: jasmonic acid

Transcription factors (TFs) play crucial roles in many biological processes, including flowering time regulation [33]. In the current study, we identified 78 genes encoding TFs among the common DEGs in leaf and shoot tissues. These genes were divided into 29 TF families, including *ERF*, *NAC*, *bHLH*, *bZIP*, and *C*_*3*_*H*_*2*_ genes, with the same expression patterns detected in both leaves and shoots (Fig. 5a). Plant hormones also help regulate flowering time [1]. In this study, 116 hormone-related genes were identified from among the common DEGs in leaf and shoot tissues (Fig. 5b). The top three such genes were related to abscisic acid (33), auxin (27), and ethylene (24).

### Functional classification of common DEGs involved in flowering time pathways

To further explore the roles of the common DEGs identified in both leaf and shoot tissues, 99 important biological pathways in *B. napus* were identified in the KEGG pathway database (Additional file 5: Table S5). Among these significant pathways, ribosome, biosynthesis of amino acids, carbon metabolism, oxidative phosphorylation, and ubiquitin-mediated proteolysis were the most highly represented pathways to which common DEGs were assigned. Important pathways including RNA transport and plant hormone signal transduction were also identified in this study (Additional file 5: Table S5).

### Expression analysis of homologous genes influencing flowering time in Arabidopsis

We identified 1172 homologs of FTR genes in the *B. napus* genome using BLASTN analysis (Additional file 6: Table S6). The *B. napus* FTR genes were classified into nine flowering-related pathways (number of genes shown in parentheses): aging (43), ambient temperature (25), circadian clock/photoperiod (401), flower development and meristem identity (58), flowering time integrator (38), autonomous (454), hormones (98), vernalization (67), and sugar pathways (46). Many genes are involved in more than one pathways (Additional file 6: Table S6).

**Fig. 6:**
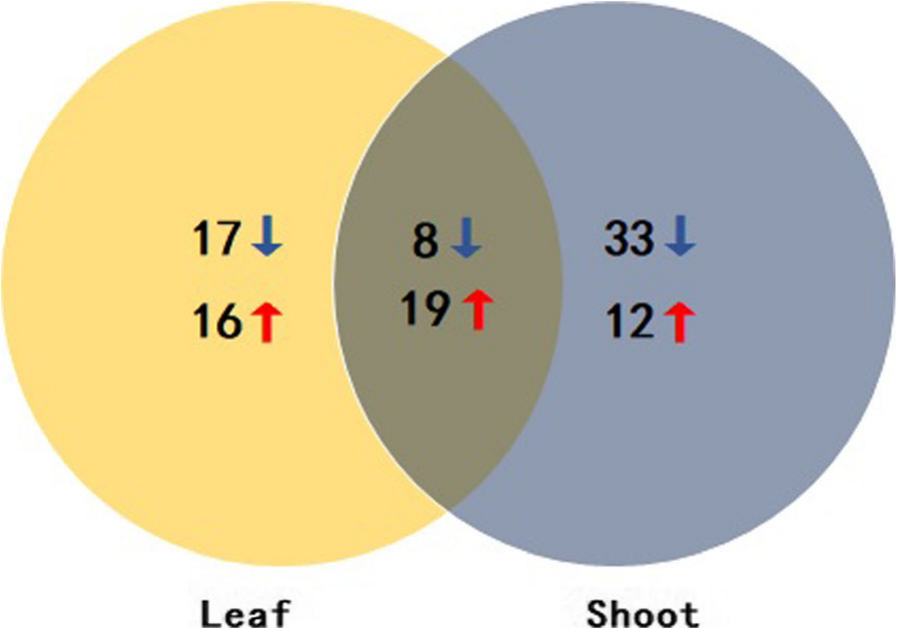
Venn diagram comparing the number of DEGs in leaf and shoot tissues between early- and late-flowering bulks. Blue and red arrows indicate downregulation and upregulation, respectively. Numbers indicate genes differentially expressed in early-flowering bulks compared to late-flowering bulks

To identify DEGs related to the flowering pathway, we screened DEGs between two bulks with extreme differences in flowering time among these putative FTR genes. In total, 105 flowering time genes were identified as DEGs using the criteria: |log2 fold change| > 0.58 (|fold change| > 1.5), FDR < 0.05 (later-flowering lines as a control) (Additional file 7: Table S7). Of these, 60 and 72 DEGs were identified in leaf and shoot tissues, respectively. Furthermore, 19 upregulated and eight downregulated DEGs were commonly identified both in leaf and shoot tissues (Fig. 6). The differentially expressed FTR genes mainly belong to the autonomous (27), circadian clock/photoperiod (38), and flower development and meristem identity pathways (14). *BnaC02g04790D*, a homolog of *FY* located in a major QTL on chromosome C02, plays crucial roles in the autonomous pathway. Genes including *FVE*, *UBIQUITIN CARRIER PROTEIN 1* (*UBC1*), *UBIQUITIN-SPECIFIC PROTEASE 13* (*UBP13*), and *LSD1-LIKE 2* (*LDL2*) were also detected, as well as an upregulated gene in the circadian clock/photoperiod pathway, *BnaC02g03470D*, encoding PRR7 (which promotes flowering). Several other important circadian clock/photoperiod pathway genes were also detected, such as *PRR3*, *PRR9*, *TIME FOR COFFEE* (*TIC*), *TIMING OF CAB EXPRESSION 1* (*TOC1*), *LATE ELONGATED HYPOCOTYL* (*LHY*), *CYCLING DOF FACTOR* (*CDF*), and *CONSTANS-LIKE 5* (*COL5*). Key genes in the flower development and meristem identity pathways were detected, including *AGAMOUS-LIKE 14* (*AGL14*), *APETALA 1/2* (*AP1/2*), and *LFY*. Many crucial genes in other pathways, such as the aging (*TOE1*), ambient temperature (*AGL31*), hormone (*GA2ox1*), sugar (*SUS4*), and vernalization pathways (*FLC* and *VIN3*), as well as flowering time integrator genes (*FLC*, *SOC1*, and *FT*) were also identified (Additional file 7: Table S7).

### Screening for candidate flowering time genes by integrating QTL mapping and RNA sequencing data

**Fig. 7:**
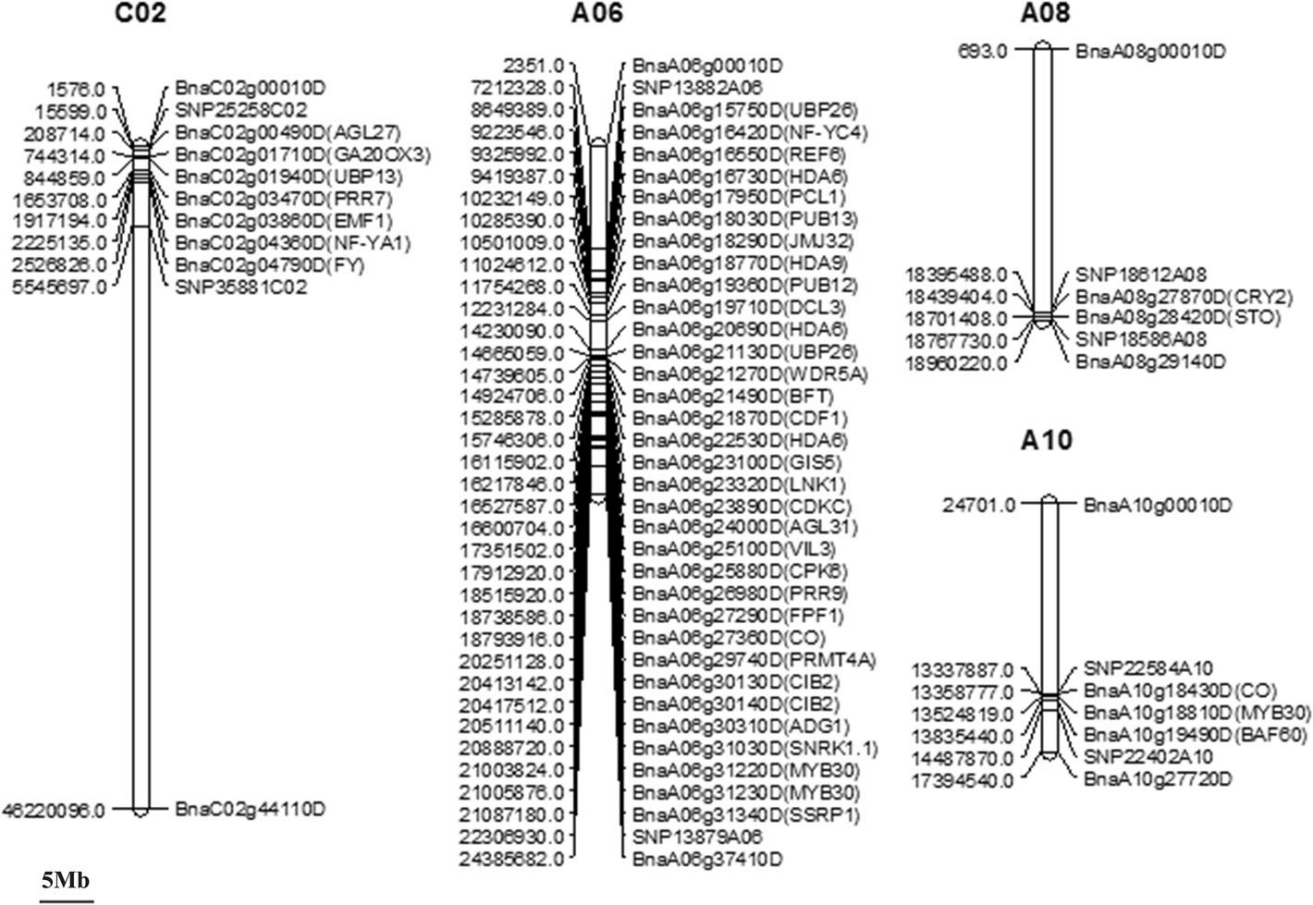
Distribution pattern of FTR genes in QTL regions. FTR genes were identified on chromosome A06, A08, A10, and C02. For simplicity, only markers in QTL confidence boundaries, along with the two terminal markers at each end of the chromosome, were shown

As mentioned above, we detected 3436 genes in QTL regions and determined their expression levels via RNA-Seq (Additional file 8: Table S8). Of these genes, 45 are FTR genes in oilseed rape (Fig. 7). Based on the criteria |log2 fold change| ≥ 1.0, FDR < 0.01 (later-flowering lines as a control), 471 genes were differentially expressed between the early- and late-flowering lines (Additional file 9: Table S9). Of these, seven flowering time-related genes were also detected (Table 5). *BnaA06g24000D*, an ortholog of *AGL31* that functions in the ambient temperature pathway, was upregulated in leaves but downregulated in shoot tissues. *BnaC02g03470D*, located in a major QTL region and encoding PRR7, plays key roles in the circadian clock pathway and was upregulated in both leaf and shoot tissues. Three autonomous pathway genes, *BnaC02g04790D*, *BnaA06g29740D*, and *BnaC02g01940D*, encoding protein transducin/WD40 repeat-like superfamily protein (FY), arginine methyltransferase 4A (PRMT4A), and ubiquitin-specific protease 13 (UBP13), respectively, were differentially expressed between the early- and late-flowering lines. These two genes function in chromatin modification and protein stability control, respectively. Of the photoperiod pathway genes, *BnaA06g16420D* and *BnaA06g30130D*, *BnaA06g16420D* was downregulated in leaves, whereas *BnaA06g30130D* was upregulated in leaves, with no mRNA detected in shoot tissues (Table 5).

**Table 5:** Seven differentially expressed FTR genes in QTL regions

Gene ID	TAIR ID	Gene	L-log2 Fold change	S-log2 Fold change	Regulator	Pathway	Conditions
BnaA06g30130D	AT5G48560	CIB2	1.310	–	Pos.	Circadian Clock/Photoperiod	LD [no data under SD]
BnaA06g16420D	AT5G63470	NF-YC4	−1.778	–	Pos.	Circadian Clock/Photoperiod	LD only
BnaA06g29740D	AT5G49020	PRMT4A	−2.060	–	Pos.	Autonomous pathway	SD and LD
BnaA06g24000D	AT5G65050	MAF2, AGL31	–	−2.916	Neg.	Ambient temperature	SD and LD
BnaC02g04790D	AT5G13480	FY	1.338	2.312	Pos.	Autonomous pathway	SD and LD
BnaC02g03470D	AT5G02810	PRR7	2.471	3.165	Pos.	Circadian Clock/Photoperiod	LD only
BnaC02g01940D	AT5G06600	UBP13	0.734	1.198	Neg.	Autonomous pathway	SD and LD

L-log2 Fold change: fold changes in leaves early- and late-flowering bulks; S-log2 Fold change: fold changes in shoots between early- and late-flowering bulks; *SD* short-day conditions, *LD* long-day conditions, *Pos.* positive, *Neg.* negative

### Verification of transcriptome sequencing data

**Fig. 8:**
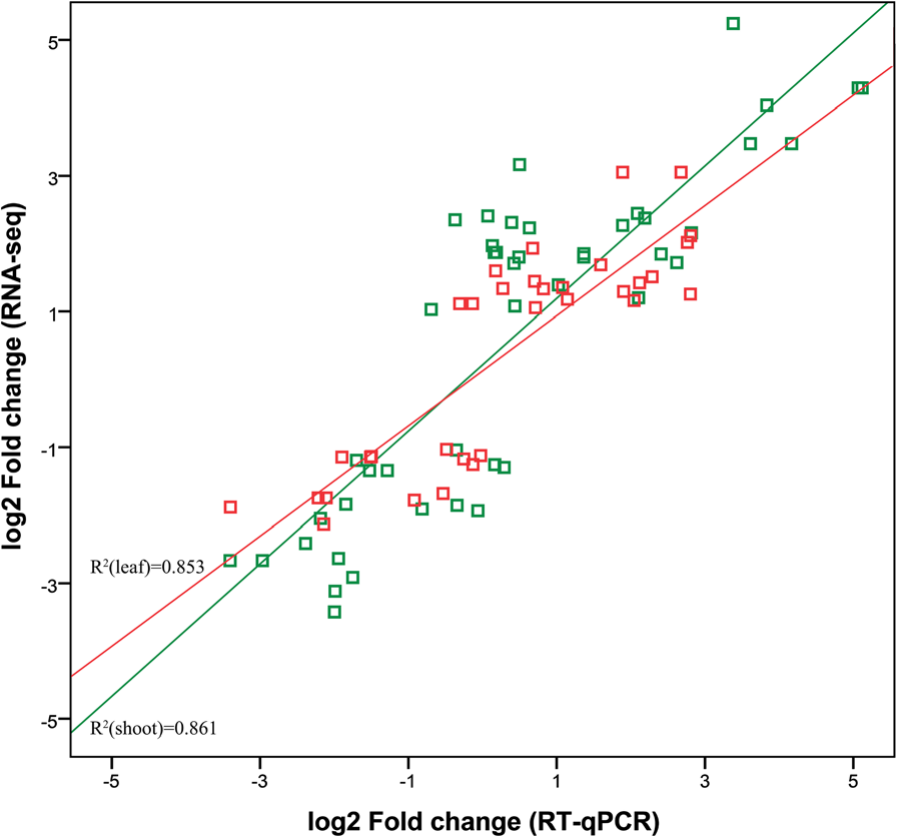
qRT-PCR validation of the expression patterns of 47 randomly selected DEGs identified by transcriptome sequencing. Red and green lines indicate regression lines for leaves and shoots, respectively

To confirm the transcriptome data and to explore selected FTR genes that were differentially expressed between the early- and late-flowering bulks, we subjected 47 randomly selected genes to qRT-PCR analysis, including TF genes, hormone-related genes, and candidate genes in QTL regions (*PRR7* and *FY*) (Additional file 2: Table S2). We detected high correlations (R^2^ = 0.853 and 0.861 in leaf and shoot tissues, respectively) between the qRT-PCR and RNA-Seq data (Fig. 8 and Additional file 10: Figure S1), suggesting that the RNA-Seq data are reliable.

## Discussion

Like many other important traits, flowering time is conditioned by the interaction of genes, endogenous signals, and environmental factors [2, 5]. In the present study, we investigated the variation in flowering time among an RIL population in six environments and in leaf and shoot tissues from early- and late-flowering lines via RNA-Seq analysis.

In the current study, 23 significant QTLs were identified under at least two conditions (Fig. 3; Table 2), suggesting that these are stable QTLs in our RIL population. Four QTLs located on chromosome A02, A05, A10, and C04 were identified under only one condition, suggesting that these QTLs are environment-specific. Flowering time QTLs located on chromosome A02, A03, A10, C02, and C03 were previously identified in *B. napus* and *B. rapa* populations [17, 34–37]. In addition, all of these regions are homologous with the top of chromosome 5 in Arabidopsis [38], a region harboring many flowering time genes such as *FLC* [39], *CO* [40], *LFY* [41], and *FY* [42]. In *B. napus*, nine homologs of *FLC* genes were detected [43]. *BnCO*s were identified on chromosome A02, A10, and C02, whereas *BnFYs* were only detected on A02 and A03 [44]. In the current study, flowering time QTLs were detected on all of the abovementioned regions except A03 and C03, and additional QTLs were identified on chromosome A05, A06, A07, A08, and C04. Finally, two QTLs located on A06 were detected in three environments, and 33 flowering time genes (e.g., *CO*, *PRR9*, and *AGL31*, Fig. 7) were identified in 7,212,328–21,686,640 (92.39–96.47 cM).

### Differential expression of *FTR* genes regulates flowering time in two contrasting bulks of RILs

In the present study, we detected important genes involved in flowering time and explored the mechanisms that regulate the flowering pathway in oilseed rape using RNA-Seq technology. We subjected leaf and shoot tissues from early- and late-flowering time lines at the vegetative stage to RNA-Seq analysis. We performed BLASTN analysis against the *B. napus* genome using 306 sequences of known FTR genes in Arabidopsis. We identified 1172 rapeseed FTR genes, 105 of which were differentially expressed between two contrasting bulks of RILs. Most of genes encoding negative regulators of flowering, such as *BnFLC*, *BnLHY*, and *BnTIC*, were downregulated in the early- versus late-flowering lines, with 35 of 51 negative regulatory genes downregulated. In addition, 22 out of 46 genes encoding positive regulators of flowering were upregulated in the early- versus late-flowering lines. The expression patterns of these genes were correlated with the corresponding phenotypes. However, there were some exceptions. For example, *BnaC03g05900D* and *BnaA02g01670D*, two orthologous genes of *FY*, were downregulated in early-flowering plants compared to late-flowering plants, whereas another *FY* ortholog, *BnaC02g04790D*, was upregulated in early-flowering plants and is located in a major QTL region. These three genes are positive regulators in the autonomous pathway, suggesting that this pathway may be partially responsible for the differences in flowering time between the two types of plants.

CDF1 (CYCLING DOF FACTOR 1) negatively regulates flowering time in Arabidopsis [45]. CDF1 suppresses the expression of *CO*, leading to the downregulation of *FT*. The expression of *FT* is positively regulated by GI (GIGANTEA) [46]. Overexpression of *CDF1* leads to later flowering, whereas the downregulation of *CDF1* (using RNAi technology) leads to early flowering under LD conditions [45]. In the current study, we identified *BnaA08g19870D* and *BnaC03g42040D* as orthologous genes of *CDF1*. *BnaA08g19870D* was downregulated in leaves, while *BnaC03g42040D* was upregulated both in leaf and shoot tissues. These results suggest that the functions of these genes differ from those in Arabidopsis.

#### Candidate genes involved in flowering time through four major pathways

FT DEGs involved in four major flowering pathways, including the circadian clock/photoperiod, autonomous, hormone, and vernalization pathways were detected. Photoperiod is an important environmental factor that regulates flowering [47]. Genes in the circadian clock/photoperiod pathway, including *LHY*, *PRR*, *CIRCADIAN CLOCK ASSOCIATED 1* (*CCA1*), *CASEIN KINASE II BETA SUBUNIT 4* (*CKB4*), *CDF*, *COL*, *CALCIUM-DEPENDENT PROTEIN KINASE 33* (*CPK33*), *AS*, and *GI* play critical roles as floral enhancers by regulating the expression of *CO* [48, 49]. Although *BnCO*s were not differentially expressed in the present study, most positive regulators in the circadian clock/photoperiod pathway, such as *PRR7*, *CPK33*, and *COL5* were upregulated in the early-flowering versus late-flowering lines, whereas negative regulators such as *LHY*, *CDF1*, and *CDF2* were downregulated. We propose that the circadian clock/photoperiod pathway is closely associated with the differences in flowering time between two contrasting bulks of RILs. However, sampling time can significantly influence gene expression involved in clock-dependent processes. In this study, we sampled in the morning, many genes such as CO and FT accumulated in the evening could not identified as DEGs.

Like the circadian clock/photoperiod pathway, the expression of autonomous pathway-associated genes corresponded with the differences in flowering time between two contrasting bulks of RILs. Key genes involved in this pathway, including *FPA*, *FY*, *FLOWERING LOCUS D* (*FLD*), *FLOWERING TIME CONTROL PROTEIN* (*FCA*), *FVE*, *FLOWERING LOCUS KH DOMAIN* (*FLK*), and *RELATIVE OF EARLY FLOWERING 6* (*REF6*), were previously characterized in Arabidopsis [50, 51]. All of the proteins encoded by these genes promote flowering by repressing *FLC* expression [52]. In the current study, we identified 27 FT DEGs involved in the autonomous pathway in oilseed rape, including *FVE, LDL2, FY, UBP13, EMF1*, and *AGL6*. Notably, *BnaC02g04790D*, a homolog of *FY* located in the major QTL region on chromosome C02, was upregulated in the early-flowering versus late-flowering lines (Table 5).

The phytohormone gibberellin promotes flowering by increasing the expression of *SOC1* [53]. Other critical genes involved in the response to GA signaling include *GID1*, *GA*, and *DELLA* [54]. In the current study, we identified ten FT DEGs in *B. napus*, including eight downregulated negative regulators of flowering. These downregulated genes include two *RGL3* genes encoding DELLA proteins and five genes encoding GA2ox1s, which are involved in the catabolism of bioactive gibberellins. Interestingly, the Arabidopsis *ga2ox1* single mutant does not display an altered flowering-time phenotype, but a quintuple *ga2ox* mutant, *ga2ox1;2;3;4;6*, flowers early under both short-day (SD) and long-day (LD) conditions [55].

Like the autonomous pathways, many genes involved in the vernalization pathway promote flowering by repressing the expression of *FLC* [56], as FLC suppresses flowering, with the help of its activator FRI [57]. Several *FLC* orthologs have been isolated and characterized in *B. rapa* [22, 58], orange [59], and *B. napus* [60]. In the present study, we detected four FTR DEGs in *B. napus* involved in the vernalization pathway, including *VRN1* and *VIN3*, encoding two components of the PRC2 complex, *WDR5A*, encoding a component of the COMPASS complex, and *AGL19*. In detail, *VRN1* and *VIN3*, encoding positive regulators of flowering time in the vernalization pathway, were upregulated in shoot tissues and leaves, respectively. *AGL19*, encoding a positive regulator of the vernalization pathway, was downregulated in shoot tissues, whereas *WDR5A*, encoding a negative regulator of this pathway, was upregulated in *B. napus*. These results suggest that the vernalization pathway may not be the main factor influencing the variation in flowering time investigated in our study.

In addition to FLC, other key floral integrators include SOC1, LFY, and FT [61]. In the current study, positive regulatory genes *SOC1* and *FT* were upregulated in both shoot tissues and leaves, while *FLC* and *LFY* were upregulated only in leaf and shoot tissues, respectively. Moreover, the key positive floral integrator gene, *LHY*, was downregulated in shoot tissues. Together, our RNA-Seq analysis identified candidate genes involved in flowering time variance in *B. napus*.

### Integration of QTL mapping and RNA-Seq results

As mentioned above, we identified 3436 genes in QTL regions, including 45 flowering time genes. We combined QTL mapping data with expression analysis of these genes via RNA-Seq. Seven FTR genes were differentially expressed in leaf or shoot tissues between two contrasting bulks of RILs (Table 5). Positive regulatory genes *BnaC02g04790D* and *BnaC02g03470D*, which are involved in the autonomous pathway and the circadian clock/photoperiod, respectively, were upregulated in both leaf and shoot tissues and are located in major QTLs on chromosome C02. *BnaC02g04790D* encodes an mRNA processing factor that regulates *FCA* expression. In addition, the expression of *FLC* is higher in *fy* single mutants than in wild type, leading to a late-flowering phenotype under both SD and LD conditions. Overexpression of *FY* in *fy* complements the mutant phenotype, leading to a normal flowering-time phenotype [42, 62, 63]. *BnaC02g03470D* encodes a component of the circadian clock in the PRR family. Functional analysis showed that the *prr7* single mutant is late flowering under LD conditions only [64, 65]. PRR7, a transcriptional repressor of *CCA1* and *LHY*, is involved in both positive and negative feedback loops of the circadian clock, thereby influencing flowering time [66]. Another positive regulator of flowering time, *BnaA06g30130D* (*CIB2*), which is involved in the circadian clock/photoperiod pathway, was upregulated in *B. napus* leaves in the current study. CIB2 is a bHLH TF that positively regulates the expression of *FT* [67]. Indeed, overexpression of *CIB2* leads to early flowering under LD [68]. *BnaA06g24000D* encodes MADS AFFECTING FLOWERING 2 (MAF2, also known as AGAMOUS-LIKE 31 [AGL31]), a negative regulator in the ambient temperature pathway. Overexpression of *MAF2* leads to late flowering under both SD and LD conditions, and the *maf2* single mutant has an early-flowering phenotype under SD and LD conditions [69–72]. MAF2 suppresses flowering in response to short cold periods [70]. *BnaA06g16420D* and *BnaA06g29740D*, encoding positive regulators of flowering, were downregulated in leaves, whereas *BnaC02g01940D*, encoding a negative regulator of flowering, was upregulated in both leaf and shoot tissues. Together, these findings highlight the complexity of the regulatory mechanisms controlling flowering time in rapeseed.

## Conclusion

In this study, we detected 27 QTLs distributed on eight chromosomes among six environments, including one major QTL on chromosome C02 that explained 11–25% of the phenotypic variation and was stably detected in all six environments. RNA-Seq analysis revealed 105 flowering time-related differentially expressed genes (DEGs) that play roles in the circadian clock/photoperiod, autonomous pathway, and hormone and vernalization pathways. We focused on DEGs related to the regulation of flowering time, especially DEGs in QTL regions. We identified 45 flowering time-related genes in these QTL regions, eight of which are DEGs, including key flowering time genes *PSEUDO RESPONSE REGULATOR 7* (*PRR7*) and *FY* (located in a major QTL region on C02). These findings provide insights into the genetic architecture of flowering time in *B. napus*.

## Additional files

Additional file 1: **Table S1.** Temperature data of each environment. Maximum and minimal temperature data of each environment in growing period were collected. (XLSX 36 kb)

Additional file 2: **Table S2.** Primers used for qRT-PCR verification. In total, 47 DEGs were selected to confirm the accuracy and reliability of RNA-Seq data used in this study. (XLSX 12 kb)

Additional file 3: **Table S3.** Correlation of flowering times among the six environments. 09Gi: Germany in 2009; 12Cq: Chongqing in 2012; 13Cq: Chongqing in 2013; 14Cq: Chongqing in 2014; 15Cq: Chongqing in 2015; 16Cq: Chongqing in 2016. ^**^Represents significance at the *P = 0.01* level. (XLSX 8 kb)

Additional file 4: **Table S4.** Genes detected in QTL regions. In total, 3436 genes were detected in QTL regions. (XLSX 199 kb)

Additional file 5: **Table S5.** Significantly enriched pathways among DEGs in leaves and shoots between early- and late-flowering bulks. In total, 100 pathways were detected among these DEGs. (XLSX 16 kb)

Additional file 6: **Table S6.** Flowering time-related (FTR) genes in *B. napus* detected using Arabidopsis FTR genes as queries via BLASTP analysis. In total, 1173 genes in *B. napus* were detected using 306 *A. thaliana* flowering time genes as queries. (XLSX 67 kb)

Additional file 7: **Table S7.** Identification of differentially expressed FTR genes in *B. napus* in leaves and shoots between early- and late-flowering bulks. In total, 105 FTR genes were differentially expressed between two bulks. (XLSX 20 kb)

Additional file 8: **Table S8.** Expression analysis of genes of QTL regions in the four samples using RNA-Seq data. EL: leaves of early-flowering bulks; LL: leaves of early-flowering bulks; ES: shoots of late-flowering bulks; LS: shoots of late-flowering bulks. (XLSX 242 kb)

Additional file 9: **Table S9.** Identification of differentially expressed genes in QTL regions. L-log2 Fold change: fold changes in leaves between early- and late-flowering bulks; S-log2 Fold change: fold changes in shoots between early- and late-flowering bulks. (XLSX 50 kb)

Additional file 10: **Figure S1.** Confirmation of RNA-Seq data using qRT-PCR technology. In total, 47 DEGs were selected to confirm the accuracy and reliability of RNA-Seq data used in this study. (TIF 192 kb)

## Abbreviations

AGL14: AGAMOUS-LIKE 14
AP1/2: APETALA 1/2
CCA1: CIRCADIAN CLOCK ASSOCIATED 1
CDF: CYCLING DOF FACTOR
CDF1: CYCLING DOF FACTOR 1
CKB4: CASEIN KINASE II BETA SUBUNIT 4
CO: CONSTANS
COL5: CONSTANS-LIKE 5
CPK33: CALCIUM-DEPENDENT PROTEIN KINASE 33
DEGs: Differentially expressed genes
DH: doubled haploid
FCA: FLOWERING TIME CONTROL PROTEIN
FLC: FLOWERING LOCUS C
FLD: FLOWERING LOCUS D
FLK: FLOWERING LOCUS KH DOMAIN
FPKM: fragments per kilobase of exon per million mapped fragments
FRI: FRIGIDA
FT: FLOWERING LOCUS T
FTR: Flowering-time related
GO: Gene Ontology
GWAS: Genome-wide associated mapping
LD: long-day
LDL2: LSD1-LIKE 2
LHY: LATE ELONGATED HYPOCOTYL
LOD: Logarithm of the odds
MAF2: MADS AFFECTING FLOWERING 2
PRR7: PSEUDO RESPONSE REGULATOR 7
PV: Phenotypic variation
QTL: Quantitative trait locus
REF6: RELATIVE OF EARLY FLOWERING 6
RIL: Recombinant inbred line
SD: Short-day
TIC: TIME FOR COFFEE
TOC1: TIMING OF CAB EXPRESSION 1
UBC1: UBIQUITIN CARRIER PROTEIN 1
UBP13: Ubiquitin-specific protease 13
UBP13: UBIQUITIN-SPECIFIC PROTEASE 13
