# Supplementary figures and images for: Joint QTL mapping and transcriptome sequencing analysis reveal candidate flowering time genes in Brassica napus L
Source: BMC Genomics. 2019 Jan 9;20:21. doi: 10.1186/s12864-018-5356-8 (PMC6325782; doi:10.1186/s12864-018-5356-8)

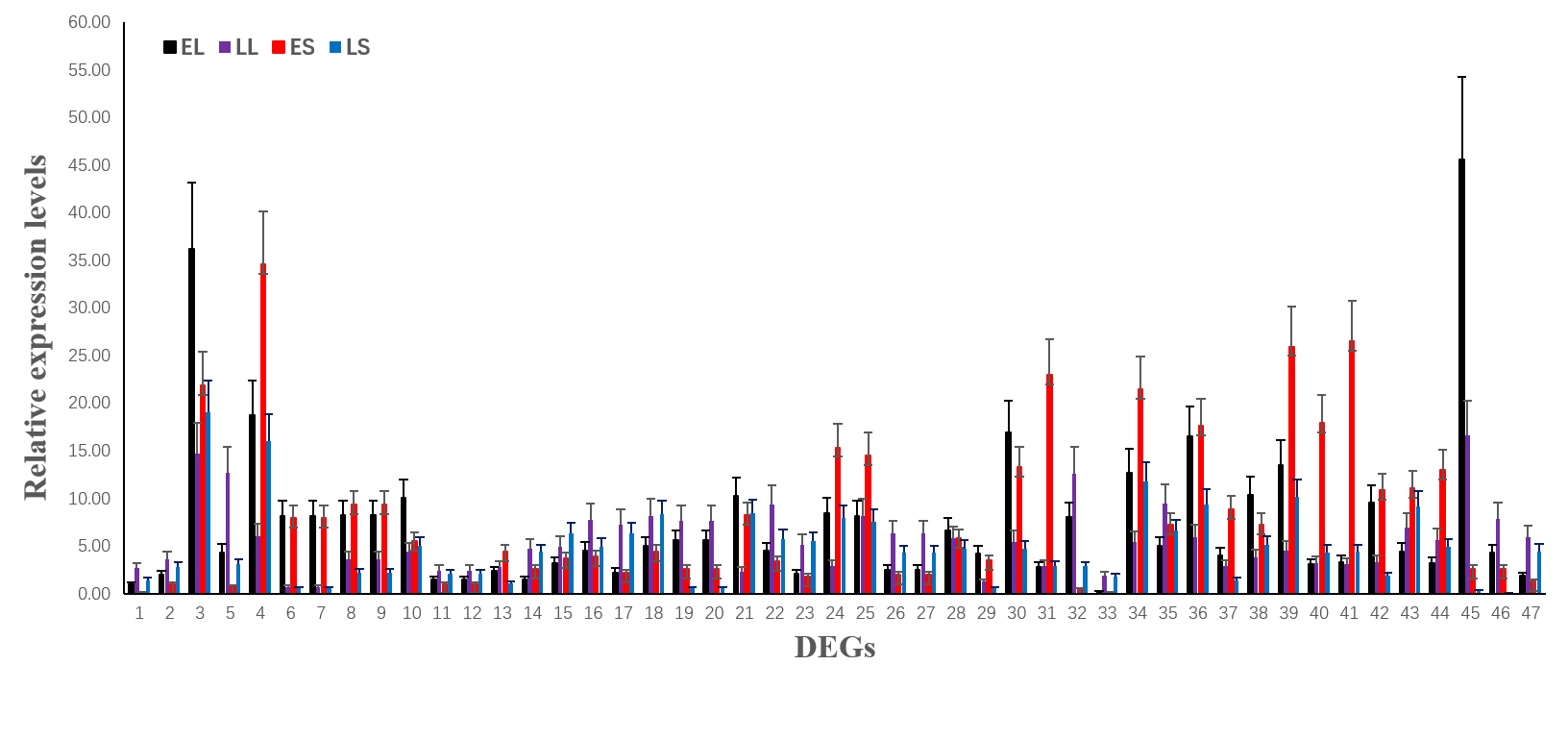

Supplement: Supplementary file 10 — Figure S1. Confirmation of RNA-Seq data using qRT-PCR technology. In total, 47 DEGs were selected to confirm the accuracy and reliability of RNA-Seq data used in this study. (TIF 192 kb) [file 12864_2018_5356_MOESM10_ESM.tif]
